# Parliament and Publications

**Published:** 1911-01-21

**Authors:** 


### Parliament and Publications.

### THE HOSPITALS OF JAMAICA.

According to the report of the Governor of Jamaica for the year ended March 31, 1910, the number of in-patients treated at the Kingston Public Hospital during that period was 3,696; the number of out-patients was 6,749, and the total cost of maintenance was £2,950 8s. 4d. The number of patients admitted into the Lying-in Hospital was 650, and the expenditure was £1,256 2s. 5d. The number of patients under treatment at the lunatic asylum during the year was 1,317. In the lepers' home at the end of the year there were 103 patients. In view of the long immunity from small-pox in the island, the Governor is inclined to think that if the money now devoted to infant vaccination were steadily applied for a few years to the isolation and treatment of all cases of yaws and syphilis the effect on the vitality of the population would be very much more valuable.—"Colonial Reports: Jamaica," Cd. 4964, price 3½d.

### HOSPITALS IN THE STRAITS SETTLEMENTS.

The number of patients treated in the hospitals of the Straits Settlements in 1909 was 30,266, of whom 1,398 were Europeans. Deaths numbered 3,635 (70 being Europeans). The rate of mortality shows a reduction, the figures being for 1908, 13.24; for 1909, 12.01 of the total cases treated. The number of inmates in the Leper Asylums increased from 417 on December 31, 1908, to 483 at the end of 1909. The number of deaths was also greater, the total for 1909 being 149. All the public hospitals of the Colony are under Government management.—"Colonial Reports," No. 663, price 4½d.

### WORK OF FOOD INSPECTORS.

That part of the annual report of the medical officer of the Local Government Board which deals with the work of inspectors of foods has been issued as a separate booklet. In seeking to apply the procedure of the Sale of Food and Drugs Acts, in the interest of the consumer, it is often difficult to stop misleading descriptions and false labelling of foods, and to repress various trade customs which result in debasing the quality or purity of staple articles of food. For general guidance in these matters a comprehensive inquiry is often required, and it is interesting to note that such an inquiry is now in progress in regard to condensed milk and the composition of foods for infants. The experimental work necessary in these inquiries has been facilitated by the provision of a chemical laboratory at the Board's offices.—"Report on the Work of Inspectors of Foods," price 4d.

### MEDICAL RELIEF OF THE POOR IN FOREIGN COUNTRIES.

Another bulky Blue Book has been issued in connection with the Royal Commission on the Poor Laws. This relates to foreign and colonial systems of relief, and contains much valuable information concerning the methods of medical relief employed abroad. In nearly all the countries cited the local relief system has had to be supplemented in the matter of medical assistance either by authorities of wider area, such as the county or the State, or by independent action on the part of the communal or municipal authority in the provision of public hospitals. The organisation created by the French law of 1893 for the free medical relief service is said to be the most complete scheme of medical relief constituted on a basis other than that of the local relief authority alone; about 2½ per cent. of the population receive medical relief in the course of the year. The law requires the *departement* to organise the medical service for its area, though the *bureau d'assistance* of the commune is responsible for granting the relief and for all dealings with individual cases. It is the business of the *departement* to map out its area into districts for hospital purposes in such a manner that each district has hospital accommodation adequate to its population, the aim being to secure one large hospital with two or three cottage hospitals to each district. For this purpose the existing Poor Law hospitals in the towns are made to serve the purpose of the neighbouring country parishes, while, where new accommodation is required, the State guarantees a subsidy towards the expenses of the *departement.* The outdoor medical service is similarly organised on a system of medical relief districts. The *departement* is responsible for the cost of medical service, though the commune pays a proportion of the cost of its own patients, and the income from endowments and other funds ordinarily accruing to the public hospitals in its district is brought into account. The State also makes a substantial grant in aid of the expenses of the *departement.* In Germany the obligation to afford medical assistance rests on the local relief authority of the commune or town; the relief authority being identical with the communal or municipal authority, the municipal authorities in the larger towns have provided hospitals; but in the country districts the construction and maintenance of hospitals has been placed within the powers of the local authority of the district, and the Poor Law authority is entitled to send sick poor to the institutions on payment. At the same time the Poor Law in Germany makes free use of charitable hospitals on similar terms. The conditions in Germany are said to be very deficient, especially in the country districts. Denmark also supplies an instance of co-operation between the relief authority and the civil authority in the provision of medical relief. In the United States medical relief is mostly left to the local relief authority, and hospital accommodation is said to be for the most part very ill-organised. As to colonial systems of medical relief, the report contains particulars concerning New Zealand, New South Wales, Victoria, Queensland, and Ontario; in all these colonies the sick poor are well provided for.—"Royal Commission on the Poor Laws and Relief of Distress," Appendix, vol. xxxiii., Cd. 5441, price 3s. 9d.

